# Factors involved in cancer metastasis: a better understanding to “seed and soil” hypothesis

**DOI:** 10.1186/s12943-017-0742-4

**Published:** 2017-12-02

**Authors:** Qiang Liu, Hongfei Zhang, Xiaoli Jiang, Caiyun Qian, Zhuoqi Liu, Daya Luo

**Affiliations:** 1 First Clinical Medical College, School of Medicine, Nanchang University, Nanchang, People’s Republic of China; 2 Queen Mary School, School of Medicine, Nanchang University, Nanchang, People’s Republic of China; 3 Department of Biochemistry and Molecular Biology, School of Basic Medical Sciences, Nanchang University, Bayi Road, No.461, 330006 Nanchang, People’s Republic of China; 4 Jiangxi Province Key Laboratory of Tumor Pathogens and Molecular Pathology, Nanchang University, Nanchang, Bayi Road, No.461, 330006 Nanchang, People’s Republic of China

**Keywords:** Cancer metastasis, Seed, Soil, Metastasis research

## Abstract

Metastasis has intrigued researchers for more than 100 years. Despite the development of technologies and therapeutic strategies, metastasis is still the major cause of cancer-related death until today. The famous “seed and soil” hypothesis is widely cited and accepted, and it still provides significant instructions in cancer research until today. To our knowledge, there are few reviews that comprehensively and correlatively focus on both the seed and soil factors involved in cancer metastasis; moreover, despite the fact that increasingly underlying mechanisms and concepts have been defined recently, previous perspectives are appealing but may be limited. Hence, we reviewed factors involved in cancer metastasis, including both seed and soil factors. By integrating new concepts with the classic hypothesis, we aim to provide a comprehensive understanding of the “seed and soil” hypothesis and to conceptualize the framework for understanding factors involved in cancer metastasis. Based on a dynamic overview of this field, we also discuss potential implications for future research and clinical therapeutic strategies.

## Background

Cancer is an important public health problem and is the major cause of human death worldwide [1]. However, tumor metastasis continues to be the main cause responsible for the cancer-related death [2, 3]. Thus, unraveling the complexity of cancer metastasis and the development of new therapeutics are imperative to restrain tumor metastasis from the primary lesion to distant organs.

In 1889, the English surgeon *Stephen Paget* proposed the “seed and soil” hypothesis after scrutinizing the autopsy records of 735 patients with fatal breast cancer [4, 5]. This hypothesis suggested that, when a plant goes to seed, its seeds are carried in all directions but can only live and grow if they fall on congenial soil. Despite the seed and soil is an appealing metaphor, it was virtually not accorded serious consideration and was challenged by *James Ewing* who declared that metastasis is determined by purely mechanical mechanisms such as anatomical and hemodynamic factors of the vascular system [6].

In recent years, additional fundamental discoveries have brought fresh insight into our understanding of cancer metastasis, and several novel concepts have been established. For example, the “tumor self-seeding” hypothesis argued that circulating tumor cells (CTCs) can seed not only to regional and distant organs in the body but also to the original source, the primary tumor itself [7, 8]. Pre-metastatic niche, conceptualized as a fertile soil conducive to the survival and outgrowth of metastatic seed, has attracted increasingly more attention in the era of metastasis research. In this review, we provide a comprehensive understanding of the “seed and soil” hypothesis, and we conceptualize the framework for understanding factors involved in cancer metastasis. More importantly, we highlight the dynamic interplay between seed and soil.

### Seed factors

**Fig. 1:**
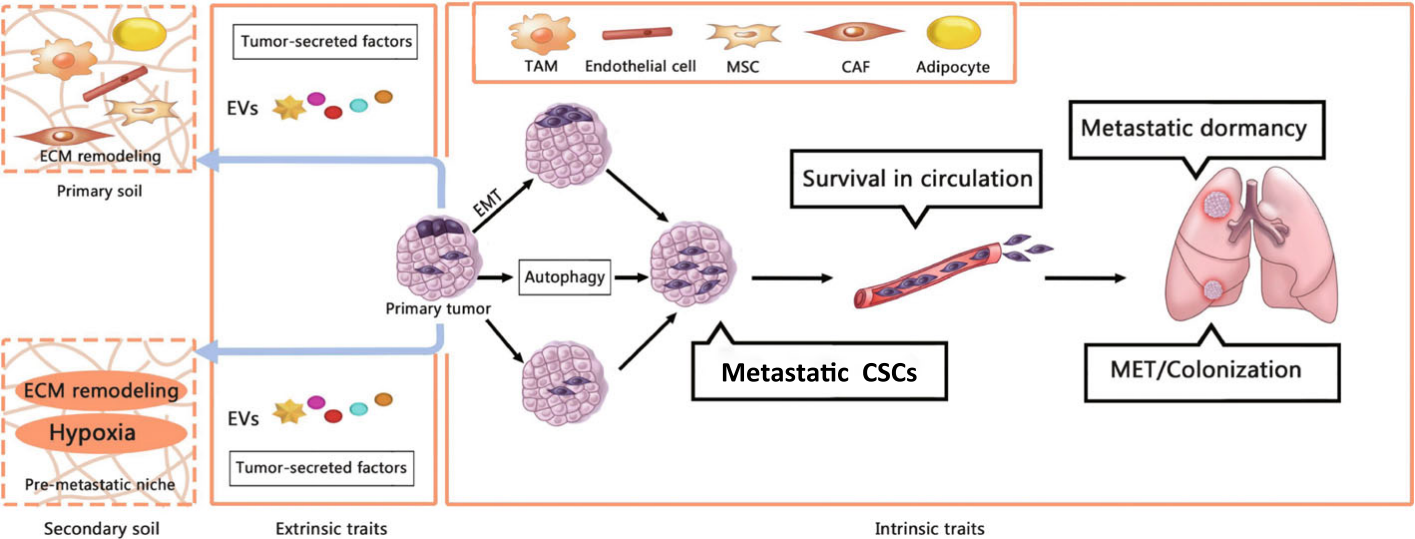
Seed Factors, both seed intrinsic and extrinsic factors are depicted here. Seed extrinsic traits remodel the primary soil and secondary soil via tumor secreted factors, inducing ECM remodeling and hypoxia, and promoting formation of pre-metastatic niche. Seed intrinsic traits, including CSC, EMT-MET, Autophagy and metastatic dormancy, is in involved in cancer metastasis, EMT and autophagy are linked with stemness of CSCs. Two alternative means of generating CSCs are depicted here, intrinsic CSCs are thought to exist in primary tumors from the very early stages of tumorigenesis and induced CSCs may arise as a consequence of EMT. CSCs with metastatic potential would be the most tenacious “seed” invasion through surrounding tissues, and intravasation, as well as survival in circulation and the eventual colonization at distant sites

Since the seed and soil hypothesis first emerged, a plethora of studies have been focused on identifying how the “seed” (cancer cell) contributes to metastasis; indeed, the seed factors (Fig. 1) play a crucial role in tumor progression and outgrowth. Herein, we provide a comprehensive review of seed factors involved in metastasis based on the latest findings and specialized articles that cover them in depth.

#### EMT-MET and metastasis

EMT (epithelial to mesenchymal transition) represents a shift toward the mesenchymal state, allowing cells to adopt migratory and invasive behavior [9], while the reverse process is referred as mesenchymal to epithelial transition (MET). EMT has been implicated in the process by which cancer cells enter the circulation and seed metastases [10]. *Yu* et al. analyzed the EMT in CTCs from breast cancer patients and found that EMT plays a critical role in the bloodborne dissemination of human breast cancer [11].

Although EMT was thought to be important in tumor progression, it is inconsistent with the fact that metastatic lesions share the epithelial nature of primary tumors [12]. To explain this apparent paradox, it was proposed that EMT is reversible [13]. Notably, there are a few studies also supporting a role for MET in distant sites. MET was implicated in the formation of clinically significant metastasis in bladder cancer [14]. In addition, accumulating experimental evidence showed the requirement of MET in the colonization and metastasis of carcinomas [15, 16], which suggests implications for future therapies against metastasis. Targeting EMT alone might be counterproductive, inhibiting both EMT and MET could be promising therapeutic strategy.

Most of the observations exploring the role of EMT in tumors have relied on cell culture-mediated loss-of-function and gain-of-function trials. However, more recently, two papers published in *Nature* provided intriguing evidence that EMT is not required for metastasis in vivo [17, 18]. While these results are interesting, researchers also suggest potential limitations to these studies, such that tracking cells based on the expression of a single gene may miss ongoing EMT events [19], and these genetic manipulations may fail to suppress the expression of versions of EMT and completely suppress activation of EMT [20, 21]. Hence, in the future, further evidence is required to support the conclusion that EMT is not required for metastatic colonization.

#### CSCs and metastasis

The cancer stem cell (CSC) theory suggests that many types of solid tumors are hierarchically organized and sustained by a distinct subpopulation of CSCs [22]. In the cancer stem cell model, cancer stem cells are described as a reservoir of cells within the tumor that have the ability to self-renew and to provide the heterogeneous lineages of cancer cells that constitute the tumor [23, 24]. Alternative term in literature is described as “tumorigenic cell”, or “tumor initiating cell”. It has been known purely a minority of cancer cells have the ability to form tumor [25, 26] and metastatic colonization is a highly inefficiency process that only a small subpopulation of disseminated tumor cells accomplished [27].

In 2005, the concept “migrating cancer stem cell” was first established, which support the existence of mobile cancer stem cell [28]. A subset of cells from human brain tumors showing stem cell properties were identified in vitro and in vivo [29], thus providing strong support for the CSC theory. In particular, this model integrated two decisive features, stemness and EMT. Evidence was provided that CSCs derived from metastatic breast tumor cells exhibit significantly higher tumorigenic and metastatic capacities than low metastatic cells [30]. Most intriguingly, the EMT program was indicated to play a pivotal role in facilitating the entrance of non-stem cells into stem cell states. For example, in a mammary tumor progression model, it was suggested that the acquisition of stem and tumorigenic property is stimulated by EMT induction [31]. Collectively, based on these observations, cancer stem cells have the capacity of long-term self-renewal and therapy-resistant, making it more possible to transform from the primary tumor, survive in the circulation and colonize distant sites. In other words, the cancer stem cell would be the most tenacious “seed” to successfully colonize in a foreign “soil”.

#### Autophagy and metastasis

Autophagy is an intracellular degradation system, by which cells breakdown cytoplasmic materials in the lysosome [32]. Although autophagy has long been postulated to be involved in cancer metastasis for many years [33], the exact role of autophagy and underlying molecular mechanisms is still controversial.

On one hand, evidence indicated that autophagy showed an anti-metastatic effect [34]. For example, *Brahma* et al. showed that rottlerin (a protein kinase C-delta inhibitor) can stimulate autophagy, resulting in cell death in pancreatic CSCs [35]. Similarly, caffeine (neuroactive compounds) induced autophagy and promoted apoptosis in various cell lines, including cancer cells. These results are consistent with previous studies on the use of caffeine to treat human tumors [36].

On the other hand, emerging experimental data support the idea that autophagy plays a pro-metastatic role in cancer metastasis, for its involvement in regulating tumor invasion [37], anoikis resistance [38], CSCs viability [39–41], EMT program [42, 43], and tumor colonization [38, 44]. Clinically, pancreatic cancers exhibit high basal levels of autophagy, and thus, a phase II clinical trial (https://clinicaltrials.gov: NCT01273805) and translational study of hydroxychloroquine (HCQ), an inhibitor of autophagy, in patients with previously treated metastatic pancreatic cancer was started. However, inconsistent autophagy inhibition was achieved and demonstrated negligible therapeutic efficacy [45]. Taken together, the role of autophagy in cancer metastasis still warrants further investigation, and clinical translation by targeting autophagy remains to be achieved.

#### Metastatic dormancy

A clinical phenomenon has been recognized for years that many patients relapse with metastatic disease months or years after primary tumor treatment because residual tumor cells can enter a dormant state and become refractory to therapies. Tumor dormancy is described as a lag time between dissemination and metastatic outgrowth, in which disseminated tumor cells (DTCs) maintain quiescence, which is a stable, non-proliferative cellular state. Tumor cells can disseminate to distant sites and enter a dormant state for long periods, only to then give rise to metastasis [46].

With regard to cancer cell itself, dormant cancer cells retrieved from metastasis-free distant sites retain their metastatic ability [47]. Recently, numerous genes have been shown to be correlated with dormancy in many types of cancer [48–50]. For example, in a bone metastasis dormancy model, VCAM-1 expression by cancer cells is shown to support reactivation of indolent cancer cells and bone metastasis by interacting with the microenvironment [50]. These evidences showed the crucial role of cancer cells in mediating tumor dormancy and reactivation.

According to experimental and clinical evidence, tumor dormant state can also be regulated by microenvironmental factors in certain organs. For instance, evidence was provided that BMP7 (bone morphogenetic protein 7), secreted by bone stromal cells, plays a key role in dormancy and recurrence of prostate cancer [51]. TGF-2β and p38 signaling was shown to keep DTCs in a dormant state in bone marrow but not in lung [52]. In a breast cancer model, the BMP inhibitor Coco was demonstrated to promote breast cancer cell dormancy escape [53].

The findings discussed above suggest that dormancy and reactivation are governed by complex interactions between DTCs and the microenvironment of the target organ. Intriguingly, it was indicated that the induction of autophagy is involved in the induction and survival of dormant ovarian cancer cells [54]. Future research requires more endeavors to find out the link between autophagy and tumor dormancy, which will provide insights into novel therapeutic strategies to metastatic cancer.

### Tumor-secreted factors

The extrinsic traits of cancer cells, tumor secreted factors also play a pivotal role in promoting cancer metastasis. These tumor-secreted factors include extracellular vesicles (EVs), cytokines and chemokines, and other molecular components. Numerous molecular components and mechanisms have been identified in past years.

#### Tumor-secreted extracellular vesicles (EVs)

EVs are secreted vesicles that include exosomes (30–100 nm diameter), microvesicles (MVs 100–1000 nm diameter), and newly identified “large oncosomes” (1–10 μm diameter) [55]. In recent years, EVs have been shown to play a critical role in mediating the interaction between tumor cells and host cells, which prepares the pre-metastatic niche for the formation of secondary sites [56]. In particular, cancer cells secrete considerably more EVs than normal cells, leading to a substantial increase in detectable EVs circulating in the blood. Increasing evidences have been shown that tumor-secreted EVs, containing DNA, RNA, proteins, and other molecule components, such as hypoxia-inducible factor-1α (HIF1α) [57, 58], are capable of mediating cell-cell communication and playing an essential role in inducing metastasis. Tumor-secreted exosomes are small membrane vesicles (30–100 nm) derived from the luminal membranes of multivesicular bodies and released into the extracellular milieu by fusion with the membrane. On one hand, tumor-secreted exosomes are shown to be involved in enhancing the metastatic traits of cancer cells and remodeling the primary microenvironment. In other words, educating the primary “soil” for a tumor-permissive microenvironment and metastasis. For instance, it was reported that breast cancer cells with increasing metastatic potential secrete exosomes that proportionally increase cell motility, which suggests that exosomes play a dynamic role in mediating metastasis [59]. MCF7 and MDA-MB-231 cells secrete exosomes that induce the differentiation of adipose-derived mesenchymal stem cells into myofibroblasts, increasing their secretion of factors such as SDF-1, VEGF, CCL5, and TGFβ, which are involved in regulating tumor progression and metastasis [60]. On the other hand, tumor-secreted exosomes are capable of educating distant “soil”. In pancreatic ductal adenocarcinomas (PDACs), macrophage migration inhibitory factor (MIF) was found to be overexpressed in PDAC-derived exosomes, and its blockade impeded liver pre-metastatic niche formation [61]. Exosomes from mouse and human organotropic cancer cells were found to preferentially fuse with resident cells at their predicted destination. Further, the uptake of tumor-derived exosomes by organ-specific cells was also shown to promote the pre-metastatic niche [62]. Consistently, small nuclear RNAs from primary tumor-derived exosomes were found to activate alveolar epithelial TLR3, leading to chemokine secretion and neutrophil infiltration [63], and to contribute to the pre-metastatic niche formation. In a recent study, elegant work by *Guoguang* and colleagues validated a novel mechanism concerning liver-tropism metastasis, that is, the remodeling of the liver microenvironment by EGFR-containing exosomes derived from tumor cells mediated gastric cancer metastasis [64]. Taken together, these studies indicate that primary tumor exosomes contribute to cancer metastasis by promoting the metastatic traits of cancer cell and, more importantly, by educating the primary soil and distant soil. Microvesicles (MVs), also known as “microparticles” [65] and/or “metastasomes” [57], owing to their ability to merge with and transfer a repertoire of bioactive molecular content (cargo) to recipient cells, are increasingly regarded as mediators of intercellular communication [58]. Accumulating evidence showed that many cancer-related cell biological processes correlate with accelerated rates of MVs secretion from tumor cells [66]. Particularly, MVs are also shown to play a crucial role in metastasis [67]. It was reported that osteopontin expressed by tumor-secreted MVs plays an important role in bone marrow-derived cell mobilization and colonization of tumors [65]. Large oncosomes (LO) are large (1–10 μm diameter) cancer-derived extracellular vesicles (EVs), originating from the shedding of membrane blebs. In human prostate cancer, it was found that LO that contains metalloproteinases, RNA, caveolin-1, and the GTPase ADP-ribosylation factor 6 and are biologically active toward tumor cells, endothelial cells, and fibroblasts, suggesting a mechanism that LO is involved during the formation of pre-metastatic niche [68].

#### Tumor-secreted cytokines and chemokines

It has been well established that cytokines and chemokines derived from cancer cells can selectively attract and activate different cell types. The diverse functions of these factors establish them as key mediators of communication between cancer cells and microenvironment. Thus, these factors are always associated with multiple aspects of cancer metastasis. To understand how cancer cells affect the tumor microenvironment, *Kim* et al. conducted a biochemical screen for macrophage-activating factors secreted by metastatic carcinomas; the results indicated that Lewis lung carcinoma produced factors that activated myeloid cells via TLR2 and that induced TNF-α secretion by myeloid cells, thus enhancing metastatic growth by preparing the pre-metastatic niche [69]. CCL2-expressing breast tumor cells engaged CCR2^+^ stromal cells of monocytic origin, including macrophages and preosteoclasts, to facilitate breast cancer metastasis to lung and bone [70], respectively. Similarly, tumor cell-secreted IL6 causes Stat3 phosphorylation in lymphatic endothelial cells (LECs), inducing CCL5 expression in LECs and accelerating triple-negative breast cancer (TNBC) cell metastasis [71]. In summary, these factors indeed play a pivotal role in promoting metastasis by preparing the formation of the pre-metastatic niche.

However, it is important to note that these factors mediate a complex interplay between various host cell types and tumor cells, and pharmacological inactivation of these molecules or their receptors to reduce metastasis should be cautiously employed. For instance, the inhibition of CCL2–CCR2 signaling blocks the recruitment of inflammatory monocytes, inhibits metastatic seeding and prolongs the survival of tumor-bearing mice [72]. However, a paper published in *Nature* reported a paradoxical effect of CCL2 in four syngeneic mouse models of metastatic breast cancer. Surprisingly, the interruption of CCL2 inhibition led to an overshoot of metastases and accelerated death [73].

#### Other molecular components

Other molecular components also play a pivotal role in mediating metastasis. For instance, in a previous study [74] with in vitro and in vivo models using MDA-MB-231 human breast cancer cells, it was demonstrated that tumor-derived osteopontin (OPN) induces mesenchymal stem cells (MSC) production of CCL5, mediating metastasis. Likely, breast cancer cell-derived tenascin C (TNC) promotes the survival and outgrowth of pulmonary micrometastases [75]. In hepatocellular carcinoma (HCC), it was found that a tumor-derived protein secretory clusterin (sCLU) contributed to HCC migration and EMT in vitro and metastasis in vivo [76]. Together, certain proteins derived from tumor cells also play a key role in mediating metastasis. Interestingly, a recent study reported that nucleotides released from the highly metastatic breast cancer cell also contribute to pre-metastatic niche formation by mediating lysyl oxidase secretion, collagen crosslinking, and monocyte recruitment [77].

#### CTCs and metastasis

As discussed above, the metastatic traits of seed indeed play a pivotal role during cancer metastasis; however, there is a big gap between the seeding cells and the formation of secondary tumor. Because most tumor dissemination occurs through the blood, CTCs that have been shed into the vasculature are of obvious interest [78]. Metastasis is a multistep process that includes local invasion by cancer cells, intravasation, arrest at a distant organ, extravasation, adapting to a new environment and colonizing distant organ, which is also regulated by the delivery and survival of CTCs in circulation and the ability of intravasation and extravasation [79].

After entering the circulation, the survival and transportation of CTCs depends on the physical interactions, mechanical forces and the microenvironment they encountered [80]. At the cellular and molecular levels, CTCs adhesion is a complex process involving dynamic cell-cell interactions. For instance, endothelial adhesion is necessary for CTCs dissemination, evidence indicated that monocytes promoted metastatic breast cancer cell adhesion to endothelium under flow [81]. By using in vitro models of vasculature, platelets have been shown to be an important ally for CTCs survival and extravasation [82]. More recently, CTCs clusters were demonstrated to greatly contribute to the spread of cancer [83]. In addition, physical trapping of CTCs and optimal circulation pattern are necessary for metastasis formation. CTCs were thought to preferentially arrest in the microvasculature that appeared to be more curved, branched, and stretched [84]. By imaging the establishment of brain metastases in vivo, capillary branching was revealed to be efficient in trapping CTCs [85]. Moreover, *Leonard Weiss* et al. suggested that metastatic colonization sites also correlated with blood flow patterns, as revealed by autopsy. Furthermore, CTCs that successfully survived and crossed the physical barrier imposed by the endothelium will eventually seed the distant organ [86].

Altogether, the metastatic cascade is driven by a sequence of both mechanical and molecular factors. Both favorable soil and suitable biomechanics are necessary for the eventual formation of a secondary tumor [87]. Additionally, it is self-evident that CTCs have great potential value for clinical applications, such as the early detection and novel treatments for metastases, as CTCs provide the possibility of targeting metastasis in real time [78].

### Soil factors (Fig. 2)

#### The primary soil factors

The primary tumor microenvironment has been known to play a pivotal role in the regulation of cancer metastasis [88]. Numerous studies have focused more on the seed-to-soil signaling events that explain the mechanism by which the seed remodels the microenvironment. However, the soil-to-seed signaling events have been largely ignored. In the past decades, data have shown that signals provided by the primary tumor microenvironment are important modulators of the capacity of tumor cells to invade, access the vasculature, and metastasize. A variety of stromal cells and other molecular components surrounding the primary tumor have been identified to provide signals to enhance the invasive properties of cancer cells in many experimental models (Table 1). In addition, hypoxia in the primary microenvironment also plays a pivotal role in the regulation of tumor metastasis.

**Fig. 2:**
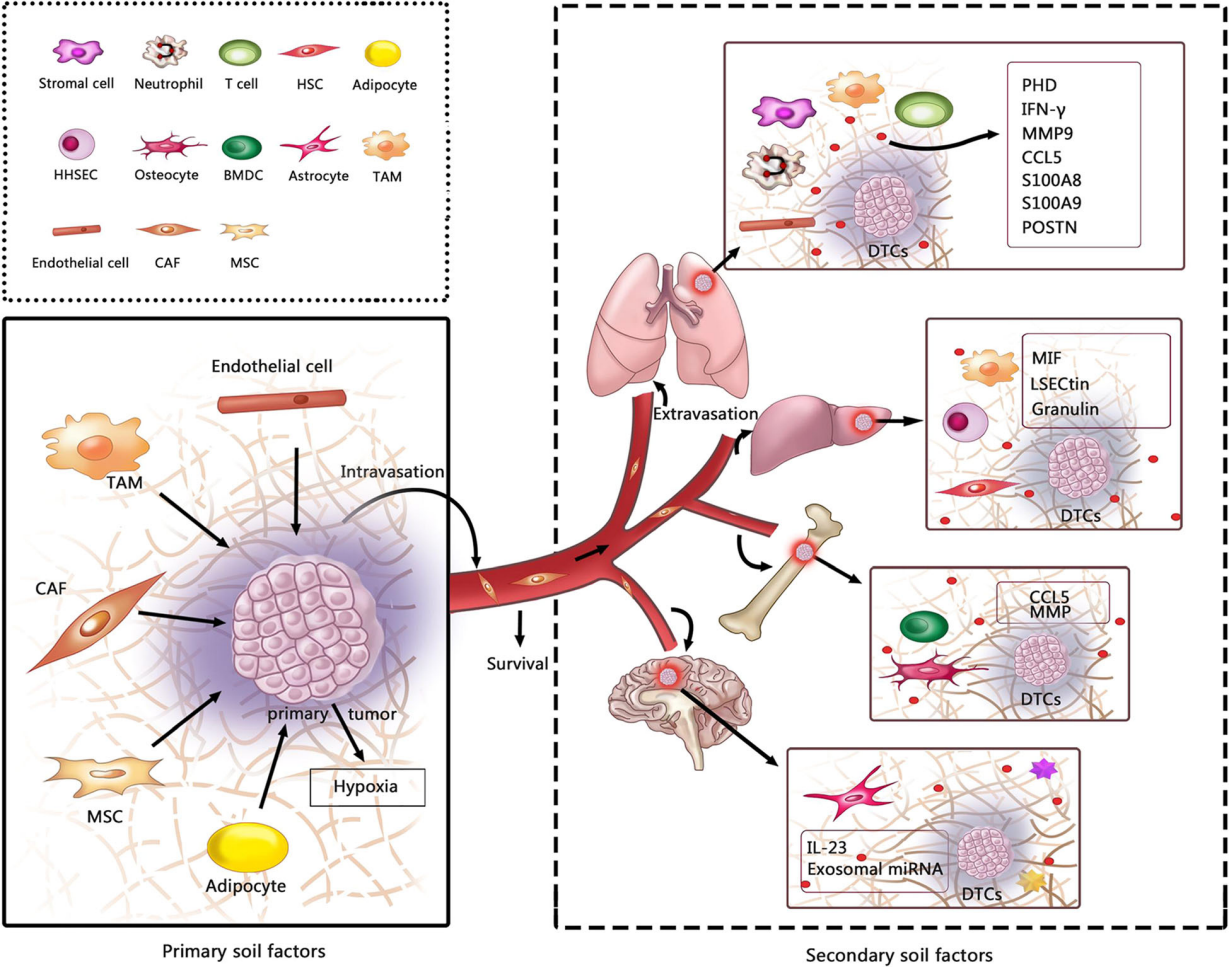
Soil factors, both primary soil factors and secondary soil factors are depicted here. Primary soil factors promote metastasis by enhancing the metastatic potential of seed. A variety of stromal cells and other molecular components surrounding the primary tumor provide signals to enhance the invasion properties of cancer cell. In addition, hypoxia in primary soil also play a crucial role in accelerating cancer metastasis. The secondary soil is composed of many factors and cell types in specific soil. The fertile cellular and molecular components in lung, liver, bone, brain, which play a pivotal role in inducing seed, invasion, eventual colonization and outgrowth at distant sites are depicted here

**Table 1:** Factors of primary soil-to-seed signaling events involved in cancer metastasis

Stroma cell type	Molecules	Underlying mechanisms	Cancer type	Ref
TAMs	miR-223	Regulates the invasiveness of cancer cells through exosome-mediated delivery of oncogenic miRNAs.	Breast cancer	[89]
	CCL18	Promotes the invasiveness of cancer cells by triggering integrin clustering and enhancing their adherence to extracellular matrix.	Breast cancer	[90]
	CCL20	CCL20 secreted by TAMs enhances the invasive properties of cancer cells via its unique receptor CCR6.	Pancreatic cancer	[91]
	Lcn2	TAM-derived Lcn2 promotes cancer cell dissemination by inducing EMT and resulting in increased cancer cell motility and invasion.	Breast cancer	[92]
MSCs	CCL5/ CCR9	MSC-derived chemokines act on cancer cells to enhance their motility, invasion and metastasis.	Breast cancer	[93, 94]
	DDR2	MSC-derived DDR2 in the primary tumor endows cancer cells with growth and migratory advantage through alignment with collagen.	Breast cancer	[95]
ECs	PHD2	Haplodeficiency of PHD2 normalizes the endothelial lining and vessel maturation, resulting in inhibited metastasis.	Melanoma Lung cancer Pancreatic cancer	[96]
	IL6	EC-derived IL-6 triggers the increased invasion of cancer cell.	Prostate cancer	[97]
	Undetermined	EC-bound tumor cells show a significantly higher anoikis resistance via the activation of Src-FAK pathway.	Squamous carcinoma	[98]
CAFs	SDF-1/CXCL12	Promote tumor growth and angiogenesis through their ability to secrete stromal cell-derived factor 1 (SDF-1).	Breast cancer	[99]
	Cav1	Stromal Cav1 remodels peri- and intratumoral microenvironments to facilitate tumor invasion, correlating with increased metastatic potency.	Breast cancer	[100]
	Exosomes	Fibroblast-secreted exosomes promote breast cancer cell protrusive activity and motility via Wnt-planar cell polarity (PCP) signaling.	Breast cancer	[101]
Adipocytes	Undetermined	Adipocytes promote tumor cell invasion and EMT program.	Melanoma	[105]
	Undetermined	Pre-adipocytes increase prostate cancer metastasis via modulation of signaling pathways.	Prostate cancer	[106]
	IGFBP-2	Adipocytes stimulate invasion of cancer cells by secreting IGFBP-2.	Breast cancer	[107]

#### Tumor-associated microphages (TAMs)

Tumor-associated macrophages (TAMs) are alternatively activated cells that are induced by interleukin-4 (IL-4)-releasing CD4^+^ T cells. It was demonstrated that TAMs enhance the invasiveness of breast cancer cells via exosome-mediated delivery of oncogenic miR-223 [89]. *Chen* et al. showed that CCL18 derived from breast TAMs promotes the invasive capacity of cancer cells by triggering integrin clustering and promoting their adherence to the extracellular matrix [90]. Similarly, in pancreatic cancer, CCL20 released from TAMs enhances the invasiveness of cancer cells via its unique receptor CCR6 [91]. More recently, TAM-secreted lipocalin-2 (Lcn2) was found to promote cancer cell dissemination by regulating EMT, resulting in increased cancer cell motility [92].

#### Mesenchymal stem cells (MSCs)

Mesenchymal stem cells have been shown to localize to breast carcinomas, where they integrate into the tumor-associated stroma. It was proven that MSCs within the tumor stroma enhance the metastatic ability of cancer cells, which is dependent on CCL5 signaling via its chemokine receptor CCR5 [93, 94]. To elucidate mechanisms involved in cancer cell migration, CCL9 produced by MSCs was also shown to play a critical role in promoting cancer cell invasion [94]. More recently, *Gonzalez* and colleagues reported that MSC-derived DDR2, a unique receptor tyrosine kinase activated by fibrillary collagen in the primary tumor, endows cancer cells with growth and migratory advantage through alignment with collagen [95].

#### Endothelial cells

PHD proteins serve as oxygen sensors and may modulate oxygen delivery, and haplodeficiency of PHD2 normalized the endothelial lining and vessel maturation, which promoted tumor perfusion and oxygenation and inhibited the migration ability of cancer cells [96]. Furthermore, it is revealed that endothelial cells within the prostate cancer microenvironment secreted IL-6, resulting in the downregulation of androgen receptor (AR) signaling in prostate cancer cells, which enhance the invasion of cancer cells [97]. More recently, it is important to note that these studies showed a novel role of endothelial cells in promoting metastasis by chaperoning circulating tumor cells and protecting them from anoikis [98].

#### Carcinoma-associated fibroblasts (CAFs)

In breast cancer, data indicated that CAFs promote tumor growth and angiogenesis through their ability to secrete SDF-1 (stromal cell-derived factor 1)in large part [99]. Further, Stroma associated with cancer metastases is enriched in Cav1-expressing CAFs, in vitro and vivo, and it was demonstrated that fibroblast expression of Cav1 (caveolin-1) favors migration and invasion of cancer cells by regulating p190RhoGAP, and stromal Cav1 remodels microenvironments to facilitate tumor invasion [100]. Intriguingly, exosomes secreted by CAFs also were shown to promote breast cancer cell protrusive invasion and motility through Wnt-planar cell polarity (PCP) signaling [101].

#### Adipocytes

Recently, increasingly more evidence supports the concept that adipocytes are associated with tumor growth and metastasis, and several molecules derived from adipocytes in the tumor microenvironment are associated with metastasis [102, 103]. Evidence also indicated that adipocytes promote cancer cell invasion [104] and the EMT program [105]. In prostate cancer, it was shown that pre-adipocytes promote metastasis by modulating miR-301a/AR/TGF-β1/Smad/MMP9 signals [106]. More recently, data indicated that IGFBP-2 secreted by mature adipocytes increases breast cancer cell invasion [107]. Together, these observations support the point that signals provided by adipocytes may play a key role in mediating metastasis.

#### Other cellular and molecular components

Other molecular signaling within the primary microenvironment can also regulate tumor metastasis by increasing the metastatic potency of tumor cells and modulating the microenvironment [108, 109]. Platelets have long been recognized as contributing to tumor metastasis [110]. Data indicated that platelet-derived TGFβ and platelet–tumor cell contacts synergistically activate signaling pathways in cancer cells, resulting in their transition to an invasive mesenchymal-like phenotype and thus promoting metastasis in vivo [111]. Similarly, adenine nucleotides derived from tumor cell-activated platelets regulate the opening of the endothelial barrier to allow transendothelial migration of tumor cells via the P2Y_2_ receptor and therefore promotes cancer cell extravasation [112]. Together, these observations suggest that signals from platelets promote several steps of tumor metastasis. By using genetic mouse models and pharmacological inhibitors, pericyte depletion was demonstrated to be associated with enhanced hypoxia, EMT and Met receptor activation, which provided evidence that pericytes within the primary tumor microenvironment likely serve as important gatekeepers against metastasis [113]. Most intriguingly, data suggest that immune cells within the primary microenvironment provide signals that promote tumor metastasis. For instance, B lymphocytes were found to be associated with tumor cell dissemination and EMT activity. More recently, infiltrating CD4^+^T cells could promote tumor metastasis by enhancing cancer cell invasion via modulation of FGF11/miRNA-541/AR/MMP9 signaling [114].

#### Hypoxia in primary soil

Because of the rapid growth of tumor cells, the tumor often outpaces its blood supply, resulting in substantial hypoxia. Hypoxia has long been recognized to promote aggressive phenotypes of tumor and induce tumor invasiveness and metastasis. To investigate the molecular mechanism of Notch-ligand activation by hypoxia in primary soil, it was found that hypoxia activates Jagged2 in breast cancer cells, induces EMT and enhances cell survival in vitro [115]. Moreover, intratumoral hypoxia promotes metastasis by activating hypoxia-inducible factors (HIFs). Data showed that HIFs regulate molecular signaling between breast cancer cells and MSCs to stimulate metastasis [116]. More recently, a paper demonstrated that the HIF-1a/CCL20/IDO axis plays a crucial role in accelerating cancer metastasis in hepatocellular carcinoma by inducing EMT and an immunosuppressive tumor microenvironment [117]. Moreover, it is worth noting that hypoxia in the primary tumor is also involved in facilitating pre-metastatic niche formation in secondary organs by providing cytokines and growth factors that create a congenial microenvironment via the recruitment of myeloid cells and a reduction in cytotoxic effector functions of the NK cell population [118].

### The secondary soil factors

It is conceivable that the “secondary soil” factors, that is, the distant organ microenvironment or the metastatic microenvironment, play a critical role in promoting colonization and metastasis outgrowth. The secondary soil is composed of many factors and cell types that influence cancer metastasis. Research to date has predominately focused on intrinsic programs of tumor cells that induce the pre-metastatic niche. However, the signals provided by distant organ remains largely unknown. Unquestionably, the preferential outgrowth of metastases in organs such as the lung, liver, bone, and brain is largely due to the presence of endogenous microenvironments within these organs that contain special metastatic niche-promoting cellular and molecular components. Herein, we summarize what is currently known regarding the key factors within secondary soil that promote tumor invasion, colonization and outgrowth (Table 2).

**Table 2:** Factors of secondary soil-to-seed signals involved in promoting metastasis

Metastatic sites	Molecules	Cell type	Underlying mechanisms	Cancer type	Ref
Lung	POSTN	Stromal cells	POSTN expressed in stroma recruits Wnt ligands and thereby increases Wnt signaling in cancer stem cells.	Breast cancer	[27]
	MMP9/ VEGFR-1TK	Macrophages Endothelial cells	MMP9 is specifically induced in pre-metastatic lung endothelial cells and macrophages, thus promoting metastasis.	Lung cancer Melanoma	[119]
	α4-integrins	Macrophages	Macrophage binding to receptor VCAM-1 in cancer cells transmits survival signals.	Breast cancer	[120]
	CCL5	Endothelial cells	CCL5 expression enhances lung colonization by recruiting innate immune cells to the metastatic microenvironment.	Colorectal cancer	[121]
	S100A8/ S100A9	Undetermined	Upregulation of chemoattractants and recruitment of myeloid cells facilitate the pre-metastatic niche formation.	Lung cancer Melanoma	[122]
	IFN-γ/ MMP9	Myeloid cells	Remodel the pre-metastatic lung into an inflammatory and proliferative environment, thus diminishes immune protection.	Breast cancer	[123]
	Versican	Myeloid Progenitor Cells	Versican induces mesenchymal to epithelial transition of metastatic cancer cells by attenuating phospho-Smad2 levels.	Breast cancer	[124]
	Leukotrienes	Neutrophils	Neutrophil-derived leukotrienes aid lung colonization by selectively expanding cancer cells with tumorigenic potential.	Mammary tumor	[125]
	PHD proteins	T cells	PHD proteins function in T cells promoting lung colonization by establishing an immunologically tolerant metastatic niche.	Melanoma	[126]
Liver	Undetermined	Hepatic stellate cells (HSCs)	HSCs play a critical role in mediating pro-metastatic niche.	Colorectal cancer	[127]
	Granulin	Metastasis-associated macrophages (MAMs)	MAMs activate resident hepatic stellate cells (hStCs) to transition into myofibroblasts, thus promoting metastasis.	PDAC	[128]
	MIF	Hepatic sinusoidal endothelial cells (HHSECs)	MIF enhances migration and EMT and facilitates proliferation and apoptotic resistance in cancer cells.	Colorectal cancer	[129]
	Angiopoietin-like 6	Undetermined	Angiopoietin-like 6 accumulates in normal vessels and interacts with the cancer cell, thus promoting colonization.	Colorectal cancer	[130]
	LSECtin	Undetermined	LSECtin expressed in liver promotes colon carcinoma cell adhesion and migration.	Colorectal cancer	[131]
Bone	Fibronectin	BMDCs	BMDCs upregulate fibronectin in resident fibroblasts, facilitating the pre-metastatic niche formation.	Lung cancer Melanoma	[132]
	N-cadherin	Osteogenic cell	Osteogenic niche activates the mTOR pathway in cancer cells, promoting bone colonization.	Breast cancer	[133]
	CCL5/MMP	Osteocytes	Upregulation of CCL5 and MMP in osteocytes promotes cancer invasion and growth.	Prostate cancer	[134]
	Extracellular ATP adenosine	Osteocytes	ATP and adenosine released by osteocytes promotes cancer cell migration, growth and metastasis.	Breast cancer	[135]
Brain	Extracellular matrix	Astrocytes	Extracellular matrix secreted by astrocyte stimulates cancer cell proliferation and EMT process.	Prostate cancer	[136]
	IL-23	Astrocytes	Astrocyte-derived molecules facilitate metastasis by enhancing invasion of cancer cell.	Melanoma	[137]
	Exosomal miRNAs	Astrocytes	Astrocyte-derived factors induce PTEN loss in cancer cells, promoting brain metastasis outgrowth.	Breast cancer	[138]

#### Lung microenvironment-derived factors

Previous studies indicated that some molecules are specially induced in pre-metastatic lung endothelial cells and microphages that transmit signals to cancer cells to promote colonization and tumor growth [119–121]. For example, in the leukocyte-rich microenvironment of the lungs, macrophage binding to breast cancer cells via the α4-integrin-VCAM-1 interaction transmits survival signals, which stimulates lung-specific metastasis [120]. Inflammatory chemo-attractants and myeloid cell recruitment have been suggested to be associated with pre-metastatic niche formation [122]. Mechanistically, myeloid cells were shown to remodel the pre-metastatic lung into an inflammatory and proliferative environment, to diminish immune protection, and to induce EMT of cancer cells [123, 124]. Stroma-derived periostin (POSTN), a component of the extracellular matrix within the secondary sites lung is shown to recruit Wnt ligands and thereby increases Wnt signaling, which enables cancer stem cell maintenance and thus promotes tumor colonization and metastasis [27]. In addition, other immune cells in the lung microenvironment are also involved in promoting metastasis. For instance, neutrophils play a key role in inflammatory responses. In a mouse breast cancer model, Stefanie et al. demonstrated that neutrophil-derived leukotrienes mediate the colonization of distant lung by selectively expanding the subpool of cancer cells that retain high metastatic potential [125]. More recently, evidence supported that T-cell-intrinsic expression of the oxygen-sensing prolyl-hydroxylase (PHD) proteins function in T cells to coordinate immunoregulatory programs within the pre-metastatic lung that are permissive to cancer metastasis [126].

#### Liver microenvironment-derived factors

Evidence indicated that hepatic stellate cells (HSCs) play a crucial role in modulating the pro-metastatic niche [127]. A recent study supported that the secretion of granulin by metastasis-associated macrophages (MAMs) stimulates resident hepatic stellate cells (hStCs) into myofibroblasts that release periostin, thus leading to a fibrotic microenvironment in the liver that sustains metastatic tumor growth [128]. Similarly, macrophage migration inhibitory factor (MIF) derived from human hepatic sinusoidal endothelial cells (HHSECs) instead of colorectal cancer (CRC) cells, induced migration and EMT and promoted proliferation and apoptotic resistance in CRC cells [129]. Additionally, angiopoietin-like 6 protein, a soluble factor accumulated in hepatic blood vessels, was found to interact with circulating cancer cells, which induced liver colonization of CRC cells [130]. Liver sinusoidal endothelial cell lectin (LSECtin) expressed in the liver microenvironment is shown to directly correlate with CRC progression, including adhesion and metastasis [131].

#### Bone microenvironment-derived factors

Bone marrow-derived hematopoietic progenitor cells that express vascular endothelial growth factor receptor 1 (VEGFR1; also known as Flt1) form cellular clusters before the arrival of tumor cells, and the expression pattern of fibronectin is shown to provide a permissive niche for incoming cancer cells [132]. Furthermore, bone colonization is induced by the osteogenic niche that is mediated by heterotypic adherens junctions including osteogenic N-cadherin and cancer-derived E-cadherin, which activate the mTOR pathway in cancer cells and consequently drive tumor progression [133]. In addition, the osteocytes within the bone microenvironment were found to promote cancer invasion and growth by secretion of CCL5, MMP and extracellular ATP and adenosine in prostate cancer and breast cancer [134, 135].

#### Brain microenvironment-derived factors

Astrocytes were suggested to be associated with brain metastasis [136]. Recently, several studies reported that astrocytes within the brain microenvironment indeed play a pivotal role in mediating metastasis. For instance, IL-23 derived from astrocytes upregulates the secretion of the matrix metalloproteinase MMP2 and enhances the metastatic potential of brain metastasizing melanoma cells [137]. In particular, elegant work by Lin Zhang et al. demonstrated that astrocytes in the brain microenvironment induced the loss of PTEN in tumor cells by secretion of exosomal miRNA, which in turn created a permissive metastatic niche for cancer cells. Mechanistically, cancer cells receive signals from the brain microenvironment that lead to an enhanced secretion of the chemokine CCL2, which recruits myeloid cells that reciprocally stimulate the outgrowth of brain metastatic cancer cells through enhanced proliferation and reduced apoptosis [138].

#### The soil supports seed and evolves together

As discussed above, both seed and soil factors play a pivotal role in mediating metastasis. The seed depends on supportive soil; however, it is important to note the dynamic interplay between seed and soil affect each other and evolve together. As a best proof, CSCs are thought to be the most tenacious seed, and TAMs are particularly abundant in “soil” among the immune cells present in the tumor site [139]. CSCs require a supportive niche to maintain a balance between self-renewal and differentiation; moreover, the dynamic interplay between CSCs and TAMs may affect the functional role and phenotype of each other [140].

Accumulating data indicated that distinct subsets of TAMs were found in different tumor microenvironments and that these TAMs were classified into two macrophage classes: M1 phenotype and M2 phenotype [141]. Furthermore, distinct subpopulations of TAMs represent distinct functional roles in the tumor microenvironment [142]. Additionally, TAMs were also shown to have the ability to regulate the adaptive immune response [143]. Increasing evidences have validated the key role of TAMs in supporting tumor growth, angiogenesis and metastasis by regulating the tumor microenvironment [144, 145]. Of note, previous studies indicated that TAMs are “educated” by myeloid-derived suppressor cells and present a distinct state of macrophage polarization, which enhances the tumor-supportive role of stromal cells. This study highlights the crosstalk between TAMs, MSCs, and tumor cells and supports the idea that TAMs within the tumor microenvironment evolve together with tumor cells. More recently, experimental data showed that host-produced histidine-rich glycoprotein inhibited tumor growth and metastasis by skewing TAMs polarization away from the M2- to a M1-like phenotype. Promising therapeutic strategies may involve targeting the crosstalk between TAMs and CSCs. However, more research is required in this area to harness the great opportunities that emerging knowledge offers. Similarly, tumor-associated neutrophils in lung cancer were shown to have the ability of polarizing to either an “N1” or “N2” phenotype that inhibits or promotes tumor progression, respectively [146].

### Clinical treatments

Although great progress has been made in the last few decades, neither the efficient prevention of metastatic tumor dissemination nor the eradication of already existing metastasis have yet to be achieved in modern cancer research. While the aim of cancer treatment in other stages is curative, the objectives in the metastatic stage are mainly palliative, then the goal turns to increase survival and symptom control [147]. Currently, agents targeting seed factors have been major components of anti-metastasis therapeutic strategies.

#### Targeting seed factors

With the goal in mind that treatment of metastatic cancer is to achieve eradication of cancer cells and hence seed factors—including unique genes expressed in tumor cells [148], factors affecting the EMT program [9], the existence of CSCs [149], autophagy [150], tumor dormancy state[151] and tumor-secreted factors that play pivotal roles in mediating metastasis are ideal targets for therapeutic interventions.

By targeting seed factors, it is likely to provide a curative effect that inhibits tumor growth and reduces metastasis. For instance, in a preclinical model, schisandrin B (Sch B), a naturally occurring dibenzo cyclooctadiene lignan with very low toxicity, could inhibit cancer metastasis by suppressing TGF-β-induced EMT of tumor cells [152]. More recently, targeting metastasis-initiating cells via fatty acid receptor CD36 resulted in almost complete inhibition of metastasis in a mouse model [153]. A humanized monoclonal antibody targeting αv integrins, which are involved in cell-to-extracellular matrix and cell-to-cell interactions, was used in a multicenter phase 1 study to inhibit prostate cancer metastasis [154]. A phase II trial of AS1411 (a novel nucleolin-targeted DNA aptamer) in metastatic renal cell carcinoma [155] has been completed, which suggests a novel way to target cancer cells at the molecular level and to improve treatment.

Although a plethora of clinical trials that targeted seed factors have had substantial successes in metastatic cancer therapy [156–158], one problem plaguing the use of therapeutics targeting seed factors is drug resistance, which is determined by not only the complexity of the genomic aberrations that tumor cells harbor but also the properties of the tumor microenvironment [159]. It is also worth noting that targeting seed factors is not always effective, for example, to determine whether metastatic cancers that overexpress Her-2 (a gene found in both normal cells and cancer cells) or CEA (a protein present mostly in cancer cells) can be treated effectively with lymphocytes (white blood cells) that have been genetically engineered to contain an anti-Her-2 protein or anti-CEA protein. Two clinical trials were conducted (https://clinicaltrials.gov:NCT00924287,NCT00923806), respectively. However, both studies were terminated due to the suboptimal side effect. In particular, two recent studies published in *Nature* demonstrated that, in early lesions before any apparent primary tumor masses were detected, a subpopulation of early cancer cells was indicated to be invasive and to be able to spread to distant organs [160, 161]. These observations remind us that targeting at seed may be less effective.

Considering the abovementioned observations, disseminated cancer cells from early and later stages have metastatic potential. Therefore, therapies targeting the seed of metastasis need to address issues of heterogeneity. Taken together, targeting seed of metastasis currently seems to be challenging and less effective. Future research focusing on uncovering the mechanisms involved in drug resistance and the complex link between EMT, CSCs, autophagy and metastatic dormancy may shed light on novel treatments that involve combined targeting of these factors.

#### Targeting the primary soil factors

Based on data presented in this review, primary soil-derived factors often confer tumor cells with the ability of invasion and tumor growth. It is likely that interventions targeting these factors will inhibit cancer metastasis. For example, a phase I study of monoclonal antibody F19 targeting a cell-surface protein of tumor stromal fibroblasts was conducted [162]. More recently, in a multicenter, randomized, placebo-controlled, phase 3 clinical trial, regorafenib—the first small-molecule multi-kinase inhibitor—was used to treat metastatic colorectal cancer by blocking various signaling pathways implicated in promoting tumor progression [163]. However, it is naive to think that individual cellular or molecular components function in isolation in a complex system. For instance, despite inhibition of CCL2 or CCR2 within the tumor microenvironment was indicated to be beneficial in inhibiting metastasis [72]. Based on this preclinical model, to determine the safety and effectiveness of blocking CCL2 and CCR2 in metastatic patients, two clinical trials have been conducted, respectively (https://clinicaltrials.gov: NCT00992186, NCT01015560). However, the results proved to be less effective possibly due to the highly complicated interaction between chemokines and chemokine receptors, as various chemokine ligands do not exclusively bind to one chemokine receptor [164].

## Conclusions

### Unifying appealing hypothesis and novel concepts

In recent years, by harnessing advancing research techniques, such as genome sequencing technology, additional fundamental discoveries have brought fresh insight into our understanding of cancer metastasis, and several novel concepts have been established. However, it is important to note that these newly established concepts or hypotheses are not mutually exclusive and have improved our understanding of cancer metastasis and have enriched the connotation of each other.

As the best example of this concept, the famous “seed and soil” hypothesis and the “mechanical mechanisms” hypothesis proposed by *James Ewing* should be integrated to better understand the factors involved in cancer metastasis. The seed and soil hypothesis states that metastatic tumor cells will metastasize to a site where the local microenvironment is favorable, just like a seed will only grow if it lands on fertile soil [4]. The mechanical mechanisms hypothesis states that metastasis is determined by the pattern of blood flow [6]. Growing evidence have indicated that both mechanical mechanisms and favorable soil play complementary roles in influencing metastatic dissemination [3, 79, 80]. More importantly, the pitfall of the seed and soil model is that only “seed” and “soil” factors are considered, while there is a big gap between metastatic seeding and the formation of secondary tumor. That is, the transportation processes for a metastatic seed travelling from the primary soil to the secondary soil are not included in the model. In reality, the mechanical mechanisms model exactly concerns more about the transportation process of metastatic seed. As such, accumulating data indicate that metastasis is a multistep process, during which metastatic cancer stem cells (seeds) travel to target organs (soil) through vessels and then colonize the soil [3].

In addition, other novel concepts such as “pre-metastatic niche”, “tumor self-seeding”, and “dormant niche” have been well established. Of note, these newly established concepts are not mutually exclusive and have enriched our understanding of metastasis. Thus, we suggest these appealing hypotheses and novel concepts should be integrated to better understand the nature of cancer metastasis, which will generate guiding significance for future research in this field.

### An equal role of seed and soil

The classic “seed and soil” hypothesis was a pivotal milestone in research of cancer metastasis, and this hypothesis introduced the concept that a permissive microenvironment is required for cancer cell colonization and metastasis formation. To date, it is without question that both “seed” and “soil” are involved in the multistep process of cancer metastasis. In other words, the interaction between cancer cells and the tumor microenvironment determined the metastasis program.

However, given the data presented in this review, our current efforts focus more on characterizing the role of the “seed”. Undoubtedly, seed factors play a critical role in promoting metastasis via their intrinsic metastatic traits, which suggests the involvement of the EMT program [9, 17, 19], the existence of cancer stem cells [149, 165, 166] autophagy [150], metastatic dormancy [151] and other intrinsic traits, and extrinsic factors of seed including tumor-secreted factors— such as extracellular vesicles [56], exosomal microRNAs [63], cytokines and chemokines [72, 73, 164], and other molecular components [75, 76]—which have been described to remodel the primary microenvironment and prime the secondary microenvironment. Together, these observations focus more on how tumor-derived factors (seed) affect the microenvironment (soil) and finally induce the formation of the pre-metastatic niche.

Although the characteristics and significance of the pre-metastatic niche, which involve the formation of metastatic niches in ectopic organs driven by the primary tumor, have been well summarized [167], it is important to consider that soil factors are largely unknown. However, the mechanism by which soil factors affect the seed is poorly characterized, as summarized in this review. Moreover, the exceptional cellular and molecular components derived from unique soil, which is composed of primary soil and secondary soil, act on cancer cells and stimulate metastasis. Primary soil-derived factors have been described as molecules derived from stroma cells, such as TAMs [89–91], MSCs [93, 94], CAFs [99–101] and endothelial cells [96–98], within the primary microenvironment that provide signals stimulating the invasion and growth of tumor. In secondary soil, factors derived from common fertile soil such as lung [27, 125, 126, 168], liver [127, 129, 131], bone [132–135] and brain [136–138] were shown to play a focal role in facilitating the metastatic potential of cancer cells and the colonization, tumor growth, and formation of the pre-metastatic niche. Hence, signals provided by the soil are associated with several processes of metastasis that play a pivotal role in mediating metastasis. Conversely, despite the importance of soil-derived factors, especially the secondary soil factors, these factors are still largely unknown.

Collectively, based on a global overview of this field, an important objective for current research is to establish the idea of considering the seed and soil equally. Therefore, we emphasize that additional investigations are required to identify the soil-derived factors involved in cancer metastasis, especially the factors derived from distant soil.

### Thinking outside the seed and soil

Undeniably, both seed and soil factors play a pivotal role in mediating cancer metastasis. However, other external factors outside of seed and soil may provide novel insights that will enable a better understanding of factors involved in tumor progression and metastasis.

In recent years, with the continuous advancements of modern surgical techniques, an increasing number of cancer patients are candidates for surgery. Accumulating experimental and clinical data have revealed that surgery is involved in tumor growth and metastasis. For instance, by performing laparotomy or mastectomy to mimic the surgery, Lee et al. showed that surgery could promote tumor growth and angiogenesis in ovarian carcinoma [169]. Removal of the primary colorectal cancer correlated with improved risk of liver metastasis. Surgery-induced inflammation may facilitate metastasis by altering the distant microenvironment. In a recent paper, it was reported that reactive oxygen species (ROS) were produced by macrophages (Kupffer cells) during surgery, which altered the ultrastructure of the liver and promoted cancer cell adhesion[170]. In clinical practices, removing primary tumors is accompanied by an exceptionally rapid metastatic outgrowth in many cases, which are in line with experimental evidence. For example, Peeters et al. showed a marked increase in proliferation and a significant decrease in apoptosis in metastatic lesions [171], which suggests that metastasis is influenced by surgical resection of primary tumor in human. Thus, considering the involvement of removing a primary tumor or metastatic lesion may provide novel insights into metastasis research.

It has been well established that commensal microbiota have an impact on tissue development and immunity [172]. In the context of cancer, commensal bacteria were shown to play a key role in modulating tumor microenvironment, which controls cancer responses to therapy [173]. Fueled by recent clinical success, cancer immunotherapy using antibodies that specifically target CTLA-4 and the PD-1/PD-L1 axis to block immune inhibitory pathways is emerging as a promising future for cancer therapy. Recently, two papers published in *Science* showed that gut microbiota are involved in cancer immunotherapy. In mice and patients, it was demonstrated that the anticancer effects of CTLA-4 blockade are dependent on the gut microbiota [174]. Similarly, combining oral administration of *Bifidobacterium* and anti–PD-L1 therapy can nearly abolish tumor outgrowth by regulating the immune response in the tumor microenvironment [175].

Food intake has been thought to be associated with a risk of death and recurrence in cancer patients. Is the dietary intake of food also involved in modulating the tumor microenvironment and metastasis? Increasing evidence supports the idea that dietary phenolic compounds play a role in inhibiting cancer invasion and metastasis [176]. Moreover, combining low carbohydrate, high protein diets and the cyclooxygenase-2 inhibitor can significantly lower the levels of metastasis [177]. Deficiency of plasminogen activator inhibitor-1 produced by the host was shown to reduce metastasis promoted by the high-fat diet. In gastric carcinoma, elevated dietary linoleic acid was reported to promote cancer cell invasion and metastasis in mice [178]. In addition, other environmental chemicals may also have an impact on perturbing the tumor microenvironment [179].

Based on abovementioned discussions, extrinsic factors outside the “seed and soil” may also play a critical role in metastasis. Metastasis is orchestrated by a complex system composed of dynamic interactions between seed (cancer cell), soil (primary soil and secondary soil) and external factors. As a consequence, manipulation of one factor of this complex system will have an impact on the other factors. The external factors such as surgery or other therapeutic interventions, such as microbiota, dietary food intake and other environmental chemicals may also have an impact on interactions between seed and soil and thus may influence cancer metastasis. The classic “seed and soil” hypothesis is appealing but may be limited. Herein, thinking outside the seed and soil, we suggest that external intervention factors should also be taken into consideration. In other words, the complex system may be viewed as a dynamically open ecosystem comprising seed factors, soil factors and external intervention factors, named “microecosystem”. Moreover, inner factors may be influenced by various external intervention factors and lead to the imbalance of the “microecosystem”. A promising therapeutic concept for future treatments is to establish the idea that recovering the “microecosystem homeostasis” with a comprehensive treatment of seed factors, soil factors and external factors. Based on this new concept, we foresee that future research focusing on how the dynamically open ecosystem influences cancer metastasis will undoubtedly provide novel insights into metastasis research and designing multimodality therapeutic strategies.

### A model for prevention and control of metastasis

It would be far better to prevent the seed dissemination from the primary site to a secondary site than to treat a patient after having metastasis. With this in mind, we aim to provide suggestions for the prevention and control of seed dissemination to distant organ based on the principle of prevention and treatment of infectious diseases, which include managing the source of infection, blocking the transmission route, and protecting the susceptible population. Clearly, the primary tumor should be the source of infection, and the transmission route of seed should include the blood and lymphatic vessel. Moreover, the susceptible population refers to frequent metastatic sites; for example, in metastatic breast cancer, the lung, liver and bone [180] may be the susceptible secondary soil.

Based on the abovementioned model, treatments of managing the source of dissemination are composed of targeting the primary tumor cells and modulating the primary microenvironment by regulating tumor cell-primary microenvironment interaction. Furthermore, regulating the transmission route should be converted to targeting the interaction between the tumor cells and endothelial cells that line the vessels to block seed dissemination, or in other words, targeting tumor cell intravasation and extravasation, which is supported by recent evidence. Evidence has been provided that different molecules, signaling pathways and circulating cells are involved in promoting tumor cell extravasation across the endothelial barrier [86]. For instance, a recent paper published in *Nature* demonstrated that the tumor cell-EC interaction facilitated extravasation and metastasis via the expression of amyloid precursor protein and death receptor 6 (DR6) by tumor cells in vitro and in vivo, which suggests that treatments targeting endothelial DR6-mediated necroptotic signaling pathways may be effective in inhibiting metastasis [181]. In the future, identification of underlying mechanisms by which cancer cells interact with ECs to promote extravasation will definitely lead to the development of new therapies to reduce metastasis. Intriguingly, recent data indicated that tumor cells implanted into the brain of nude mice spread along the abluminal surface of blood vessels instead of the bloodstream [182], which raises the possibility and likelihood of an alternative mechanism of dissemination. If other pathways besides the blood and lymphatic vessels for tumor dissemination are validated, then current therapeutics targeting cancer cells in circulation may be less effective.

In addition, protecting the susceptible secondary soil should be converted to targeting organs by regulating the interaction between metastatic cancer cells and the distant organ microenvironment. A salient feature of cancer metastasis is organotrophic, as certain types of cancer tend to metastasize to specific organs. For instance, the most common sites for breast cancer metastatic spreads are bone, liver and lung [180], and bone is the major site for prostate cancer metastasis [183]. Moreover, the metastatic colonization is highly inefficient, and organ infiltration is not sufficient for metastatic outgrowth [3]. Taking a comprehensive consideration of these two traits, it would be valuable to determine the underlying molecular mechanisms involved in organ-specific colonization, which would provide support that intervention of the colonization process by targeting the secondary soil may be promising. Secondary soil-derived factors transfer signals to cancer cells that promote tumor cell colonization in the distant organ; interventions that target blocking these signals may be effective in inhibiting metastasis. Consistently, the results of a phase 3, randomized, placebo-controlled trial showed that targeting the bone microenvironment can delay bone metastasis in men with prostate cancer, which supports the idea that soil factors can be promising therapeutic targets [184].

Research on the mechanisms that soil-derived factors support tumor distant metastasis and the mechanisms underlying the pathway of seed dissemination should yield clues for innovative treatments of metastatic cancer, and making use of the prevention and control model in preventing metastasis may be promising in the future.

### Recent technology advances that favoring metastasis research

Research on cancer metastasis has been hindered by the complex biological nature of both seed and soil. In the past few decades, advancements in mass spectrometry, microarray technology, and advanced genome sequencing technology have dramatically accelerated the endeavor to comprehensively characterize the role of metastatic cancer cells in mediating metastatic disease and the relationship between primary and secondary tumors. Recently, increasingly more powerful technologies have been developed to aid metastasis research, which will definitely help us to resolve numerous important questions in this field, such as the complex interactions between seed and soil, the establishment of the pre-metastatic niche, the colonization process and the tumor dormancy state.

In particular, single-cell sequencing has emerged as a powerful technology to characterize the nature of individual cancer cells instead of analyzing bulk tissue samples composed of millions of cells [185] and has provided new insights into our understanding of the complex multicellular ecosystem of metastatic cancer [186]. The development of intravital microscopy and imaging technology has enabled the visualization and analysis of cancer cell dynamics in live animals in real time, which may therefore lead to novel findings in metastasis research and may be promising tools in designing therapeutic interventions [187, 188]. By counting methylated haplotypes within informative genomic regions, the presence of cancer cells and the tissues or organs with tumor growth can be mapped [189].

In addition, considering the complexity of the soil, characterizing single components may be insufficient to uncover the integrated role of the soil. Recent techniques employ three-dimensional (3D) culture models to reconstitute features of organs and enable in vitro recapitulation of in vivo function, which may be highly promising in accelerating the characterization of metastasis and the development of therapeutic strategies targeting the soil. Several organs have been reconstructed in vitro system, such as the lung [190, 191], liver [192] and brain [193]. Collectively, future comprehensive applications of these advanced technologies will undoubtedly facilitate our deeper understanding of the cellular and molecular mechanisms involved in the whole process of metastatic disease progression.

## Abbreviations

CAFs: Carcinoma-associated fibroblasts
Cav1: Caveolin-1
CRC: Colorectal cancer
CSC: Cancer stem cell
CTCs: Circulating tumor cells
DTCs: Disseminated tumor cells
EMT: Epithelial to mesenchymal transition
EVs: Extracellular vesicles
HCC: Hepatocellular carcinoma
HHSECs: Human hepatic sinusoidal endothelial cells
HIF1α: Hypoxia-inducible factor-1α
hStCs: Hepatic stellate cells
Lcn2: Lipocalin-2
LECs: Lymphatic endothelial cells
LSECtin: Liver sinusoidal endothelial cell lectin
MAMs: Metastasis-associated macrophages
MET: Mesenchymal to epithelial transition
MIF: Macrophage migration inhibitory factor
MSC: Mesenchymal stem cell
MVs: Microvesicles
OPN: Osteopontin
PCP: Planar cell polarity
PDACs: Pancreatic ductal adenocarcinomas
POSTN: Periostin
sCLU: Secretory clusterin
SDF-1: Stromal cell-derived factor 1
TAMs: Tumor-associated macrophages
TNBC: Triple-negative breast cancer
TNC: Tenascin C
VEGFR1: Vascular endothelial growth factor receptor 1
